# Outcome and Prognostic Factors for Traumatic Endophthalmitis over a 5-Year Period

**DOI:** 10.1155/2014/747015

**Published:** 2014-09-15

**Authors:** Simona Delia Nicoară, Iulian Irimescu, Tudor Călinici, Cristina Cristian

**Affiliations:** ^1^Department of Ophthalmology, Faculty of Medicine, “Iuliu Haţieganu” University of Medicine and Pharmacy, 8 Victor Babes, Street, 400012 Cluj-Napoca, Romania; ^2^Department of Neuroscience, Faculty of Medicine, “Iuliu Haţieganu” University of Medicine and Pharmacy, 8 Victor Babes, Street, 400012 Cluj-Napoca, Romania; ^3^Department of Medical Informatics and Biostatistics, Faculty of Medicine, “Iuliu Haţieganu” University of Medicine and Pharmacy, 8 Victor Babes, Street, 400012 Cluj-Napoca, Romania

## Abstract

*Purpose*. To evaluate the outcome and identify the prognostic factors of traumatic endophthalmitis over a 5-year period. *Methods*. We reviewed the medical records of all the traumatic endophthalmities that we treated in our department over the last 5 years (2009–2013). We extracted the following parameters: age, gender, wound anatomy, associated ocular lesions, treatment, and initial and final visual acuities. We used the program SPSS version 20.0.0. for the statistical analysis of our data. *Results*. During the last 5 years, we treated 14 traumatic endophthalmities, representing 46.66% of all types of endophthalmities. The infection rate in open globe injuries was 8.13% and 34.78%, if an intraocular foreign body (IOFB) was associated. All the patients were males with the median age of 37 years. Initial visual acuities varied between light perception and 0.4 and the timing of treatment from a few hours to 10 days. We administered antibiotic and anti-inflammatory drugs, systemically and intravitreally, in all cases. We performed pars plana vitrectomy in 64.28% of cases. In 57.14% of cases, the final visual acuity was 0.1 or more. *Conclusions*. IOFBs increased significantly the risk for endophthalmitis. The worse prognostic factors were retinal detachment at presentation and delayed treatment. This trial is registered with IRCT2014082918966N1.

## 1. Introduction

Endophthalmitis is an uncommon, but severe complication of ocular trauma [1]. The visual prognosis in an eye with traumatic endophthalmitis is very poor, much worse than in an eye with postoperative endophthalmitis, because the physical effect of the trauma itself has a major consequence on the visual function [1]. Also, the pathogens that cause traumatic endophthalmitis are distinct and more virulent than those involved in other types of ocular infection [1, 2].

The course of traumatic endophthalmitis is influenced by various factors: type of injury, microorganisms involved, association or not of IOFBs and of the retinal detachment (RD), and time from trauma to treatment [1]. If we consider that traumatic endophthalmitis touches mainly the working-age population, the interest for this condition is justified. The purpose of this study is to evaluate the outcome of traumatic endophthalmitis and to identify the prognostic factors, as resulting from our own experience.

## 2. Materials and Methods

### 2.1. Setting

This study was undertaken in the Department of Ophthalmology, belonging to the “Iuliu Haţieganu” University of Medicine and Pharmacy from Cluj-Napoca, Romania. The patients were enrolled in the study after they signed the informed consent form. The Ethics Committee of the “Iuliu Haţieganu” University of Medicine and Pharmacy approved the study.

### 2.2. Study Sample

All the consecutive patients who were diagnosed with traumatic endophthalmitis between January 1st 2009 and December 31st 2013 were included in the study. The sampling method is longitudinal retrospective. Traumatic endophthalmitis was diagnosed clinically, according to symptoms (eye pain, decreased visual acuity, photophobia, and tearing) and signs (purulent exudate at the site of injury, eyelid edema, chemosis, hypopyon, fibrin in the anterior chamber, and vitreous haze/opacification), in a patient with previous ocular trauma.

### 2.3. Medical Intervention

In all the cases, immediate systemic antibiotic and anti-inflammatory therapy was initiated.

Locally, the medical intervention consisted either in plana vitrectomy (PPV) at the end of which intravitreal injections of antibiotics and steroids were administered or intravitreal injections alone, according to the precocity of the intervention and the severity of the disease.

In all cases, we collected vitreous samples. In the nonvitrectomized eyes, the sampling was performed by vitreous needle tap. If vitrectomy was planned, we first obtained a vitreous sample of 0.2–0.5 mL without infusion, using gentle manual aspiration into a syringe with a high cutting rate. The specimen was injected within minutes deeply into liquid media and sent to the microbiology laboratory immediately. We performed Gram stain and culture for aerobic, anaerobic, and fungi, followed by the testing of antibiotic sensitivities. The specimens were inoculated within 30 minutes of collection on blood agar, chocolate agar, Sabouraud agar, and thioglycolate broth. Chocolate, blood, and thioglycolate specimens were incubated for 18 to 24 hours in a CO_2_ incubator at 35.5°C. Growth on two or more media or confluent growth on at least one solid medium at the inoculation site was considered as positive culture results. Any plate showing pathogens was tested by the minimum inhibitory concentration (MIC) method for drug sensitivity.

### 2.4. Statistical Analysis

The statistical analysis of our data was performed with the program SPSS version 20.0.0 (Chicago, IL, USA). There were calculated frequencies for the following parameters: age, gender, the association or not of an IOFB, wound anatomy, the associated ocular lesions (direct traumatic cataract, retinal detachment, and ocular tissue prolapse), initial visual acuity, culture (positive or negative), the interval between the trauma and the treatment initiation, treatment (intravitreal injections alone or combined with PPV), and final visual acuity. The parameter according to which the outcome was defined was the final visual acuity: <0.1 or ≥0.1. In every case, we considered as final, the visual acuity tested at the last visit. Statistical analyses were used to compare treatment outcomes among the study groups. Chi-square test was used to calculate the association between the categorical variables. Value <0.05 was considered statistically significant. In case of unequal data distribution, the *P* value was given by Fisher's exact test.

## 3. Results

During the last 5 years, we treated 14 cases with traumatic endophthalmitis, representing 46.66% of the 30 intraocular infections that we managed within this time frame. Over the same period, we treated 172 open globe injuries, meaning that the overall traumatic endophthalmitis rate was 8.13%. All the 14 patients were males and had ages comprised between 18 and 64 years, with a median of 37 years (±7.08). The age was below 50 years in 10 patients (71.42%) and between 60 and 64 years in 4 patients (28.57%). The ocular signs are summarized in Table 1.

Culture was positive in 9 cases (64.28%) and negative in 5 cases (35.71%). All the 9 culture-positive endophthalmities were infected by Gram-positive germs as follows:* Staphylococcus epidermis*, 4 cases (44.44%);* Staphylococcus aureus*, 2 cases (22.22%); polymicrobial, 2 cases (22.22%); and* Bacillus cereus*, 1 case (11.11%).

The interval of time between endophthalmitis diagnosis and the initiation of treatment varied between a few hours and 10 days, with the distribution illustrated in Table 2.

In order to express the visual acuities, we used the decimal system. Initial visual acuities varied between light perception and 0.4, as illustrated in Table 3.

The systemic treatment consisted in the administration of oral moxifloxacin 400 mg every 24 hours and anti-inflammatory drugs: steroidal in 11 cases (78.57%) and nonsteroidal in 3 cases (21.42%). Locally, we performed intravitreal injections of antibiotics and steroids in all the 14 cases: in 5 cases without PPV (35.71%) and in 9 cases at the end of PPV (64.28%). The combination of drugs that we injected intravitreally in every case consisted in vancomycin 1 mg/0.1 cc, ceftazidime 2.2 mg/0.1 cc, and dexamethasone 0.4 mg/0.1 cc.

Table 3 synthesizes the most important data on our series.

The length of followup varied between 1 and 15 months, with a mean of 6.21 months, as shown in Table 3. In the 6 cases, with no potential for visual acuity improvement (final VA < 0.1), the followup did not exceed 3 months after the acute episode (mean: 2.33 months). In the 8 cases, with final VA ≥ 0.1, the mean followup was 9.12 months. In 5 of the 8 cases that recovered final VA ≥ 0.1, the visual gain was obtained within the first month after the acute episode and maintained throughout the followup period. The mean followup for these 5 cases was 9.4 months. In the remaining 3 patients (cases  2, 4, and 9), the visual acuity decreased after the initial gain, subsequently to cataract development. The mean followup for these 3 cases was 8.66 months.

The association between various factors and the final visual acuity is illustrated in Table 4.

## 4. Discussion

### 4.1. Epidemiological Data

Trauma was responsible for 46.66% of all the endophthalmities that we treated during the last 5 years. This is a higher rate as compared to the ones quoted in the literature (25–30%) [1] that could partly be explained, by the fact that we are a tertiary care center, where severe cases are referred. The traumatic endophthalmitis rate within the open globe injuries group (8.13%) matches the data we found in the literature: 4–8% [3]. The presence of an IOFB increases the risk for endophthalmitis [1, 3]. This observation is confirmed by our series. Thus, during the same period, we treated 23 IOFBs, of which 8 were complicated by endophthalmitis (34.78%), a higher rate as compared to the literature: 6.9–30% [3]. Statistically, the association of an IOFB increased significantly the risk for endophthalmitis on our series (*P* = 0.03).

### 4.2. Considerations on IOFBs

IOFBs were associated in 8 of the 14 traumatic endophthalmities, representing 57.14% of cases. The composition of the IOFB influences the risk of endophthalmitis, and nonmetallic foreign bodies have a higher risk of endophthalmitis, as compared to the metallic ones [1]. On our series, 7 IOFBs were metallic and magnetic, and 1 was a glass fragment. Essex et al. reported that 8.7% of metal IOFBs developed endophthalmitis [4], a much lower figure than ours (31.81%). If the IOFB is located in the anterior segment and the lens is intact, the prognosis is better [3]. The glass fragment was the only IOFB located in the anterior segment on our series. Metallic foreign bodies develop higher speeds and therefore penetrate deeper into the eye. The association of endophthalmitis with an IOFB adds challenge to the case, as the visualization and removal of the IOFB are more difficult [3]. Of the 8 endophthalmities with IOFBs on our series, the final visual acuity was <0.1 in 5 cases (62.5%) and ≥0.1 in 3 cases (37.5%). In the 6 cases without IOFB, the final visual acuity was <0.1 in 1 case (16.66%) and ≥0.1 in 5 cases (83.33%). Statistically, significance is not reached (*P* = 0.086). However, all the three cases with very low visual acuity (no light perception, hand motion, and counting fingers at 4 inches) were in the IOFB group. Traditionally, if an IOFB is present, prompt surgery is recommended, but recent publications prove that immediate IOFB removal may not be as important as previously thought [3]. Colyer et al. and Ehlers et al. reported large retrospective series focusing on the IOFB management and outcomes and showed that there was no significant correlation between time to IOFB removal and visual acuity [5, 6]. However, an observation has to be made: the above-mentioned studies referred to ocular injuries produced by high velocity foreign bodies that may have self-sterilized before penetrating the eye [1]. On our series, 5 of the 8 IOFBs were extracted from the eye within 48 hours and 3 after seven days or more.

### 4.3. Associated Lesions

The wound was corneal in 8 cases (57.14%) and scleral in 6 cases (42.85%). The length of the wound was comprised between 1 and 8 mm. Corneal wounds can be associated with direct traumatic cataract and scleral wounds, with prolapse of the uvea and vitreous. Zhang et al. showed that posterior scleral wounds have a lesser risk of endophthalmitis as compared to corneal wounds [7]. On our traumatic endophthalmitis series, the number of corneal wounds exceeds the number of scleral wounds (8 versus 6), but the number of cases is too low to support this theory. The larger than 4 mm wounds that have a posterior location are associated with worse visual prognosis [1]. More studies are needed in order to draw significant conclusions regarding the risk of endophthalmitis related to wound site and anatomy [1]. The visual outcome was not influenced by the wound anatomy (location and size) on our series (*P* > 0.05).

Direct traumatic cataract was present in 5 cases (35.71%), ocular tissue prolapse in 4 cases (28.57%), and retinal detachment in 3 cases (21.42%) (Table 1).

There are several hypotheses explaining the increased risk of endophthalmitis in the cases with lens disruption. Lens breach gives bacteria direct access to the vitreous. It also burdens the flow of the aqueous humor, decreasing the clearance of harmful microorganisms. The germs use the lens material for nutrition and growth [1]. The association of direct traumatic cataract did not influence the outcome of traumatic endophthalmitis on our series (*P* > 0.05).

The prolapse of ocular tissue through the open wound increases the risk of endophthalmitis, by enhancing the exposure to infecting organisms. Besides, the posterior location makes the wound closure difficult and incomplete, facilitating the intraocular penetration of germs. On the other hand, a study performed by Zhang and coworkers proved the protective effect of uveal prolapse regarding the risk of endophthalmitis [7]. The prolapsed tissue plugs the wound, preventing the entry of infecting organisms. This observation is in contradiction with the study reported by Soheilian and coworkers in which vitreous prolapse was associated with a higher risk of endophthalmitis [8]. Gupta and associates showed no effect of ocular tissue prolapse on the risk of endophthalmitis [9]. In regard to the prognosis of endophthalmitis, tissue prolapse did not influence the outcome on our series (*P* > 0.05).

The condition of the retina is a very important factor that determines the visual outcome of an eye with posttraumatic endophthalmitis. The association of pus in the vitreous cavity with retinal break(s) or retinal detachment at presentation carries a very poor prognosis [1]. Thus, Brinton and coworkers reported visual acuities between no light perception (NLP) and 3/200 in these circumstances, whereas in the eyes without retinal detachment, a visual acuity of 20/200 or better was achieved in 72% of cases [10]. In another series, reported by Affeldt and coworkers, all the endophthalmitic eyes with retinal detachment developed phthisis bulbi or were enucleated [11]. The poor outcome of retinal detachment concurrent with endophthalmitis is determined by the retinal necrosis and delayed healing [1]. On our series, the association of retinal detachment (3 cases) determined a significantly worse prognosis of endophthalmitis (*P* = 0.024). Thus, all the three cases had final visual acuities below 0.1: no light perception, hand motion and 0.06. Also, in all the 3 cases, IOFBs were present.

### 4.4. Initial Visual Acuities

There is evidence that endophthalmitis cases with better visual acuities at presentation have better visual outcome [1]. We cannot confirm this observation according to our findings (*P* > 0.05).

### 4.5. Culture

The cases of traumatic endophthalmitis can be culture-independent or culture-positive. The positive culture in an open-globe injury is not synonymous with the development of posttraumatic endophthalmitis. The culture-negative endophthalmities have higher chances for visual acuity improvement [1]. This statement is not confirmed by our series: the statistical analysis demonstrates that culture-negative cases did not have a better outcome as compared to the culture-positive ones (*P* > 0.05).

About 75% of all the culture-positive posttraumatic endophthalmities are infected by Gram-positive organisms [1]. All our traumatic endophthalmities were infected by Gram-positive germs. We noted the most frequently* Staphylococcus epidermis*, 44.44%, confirming the data in the literature [1]. In one case,* Bacillus cereus* was identified. In this case, soil contamination was noted, which is a known risk factor for the infection with this germ. The case had a bad outcome, with loss of vision. In this case, timing was also inappropriate, as the patient got to our hospital 10 days after a penetrating trauma with IOFB. The highest likelihood for a bad outcome is associated with the following organisms:* Bacillus cereus*, Gram-negative rods, and fungi [1].

### 4.6. Timing

Immediate intervention is crucial for arresting the potentially destructive inflammatory response. Delayed primary repair, especially more than 24 hours, is considered to be a risk factor for the development of endophthalmitis [1]. Also, the initiation of systemic antibiotic therapy after more than 24 hours from the traumatism is associated with a significantly higher risk of endophthalmitis, as proved by Schmidseder et al. [12]. The damage comes not only from the toxic factors produced by the organism in the eye but also from the influx of inflammatory cells into the posterior segment.

In many circumstances, the visual loss associated with the trauma itself, combined with the referral of the patient from another hospital, has made it difficult for us to indicate the precise moment for the debut of endophthalmitis.

On our series, in 6 of the 14 cases (42.85%), the treatment was initiated within 24 hours. In 4 of them, visual acuity recovered to 0.1 or more (66.66%). In case  6 (Table 3), the gravity of the associated lesions in the anterior segment (large corneal wound) prevented the satisfactory recovery of visual acuity. In this case, the IOFB (glass fragment) was located in the anterior chamber and therefore PPV was not performed. In case  7 (Table 3), despite the prompt intervention by PPV, the gravity of the associated injuries (IOFB and retinal detachment) did not allow the vision recovery. All the 4 cases, in which treatment was initiated after more than 24 hours, but within 72 hours, recovered visual acuities of 0.1 or more. In none of these cases retinal detachment was associated, but IOFBs were present in 2 of them. All of the 4 cases in which treatment was initiated more than 72 hours after the trauma ended up legally blind. In 2 of them, PPV could not be carried out, because of the opaque cornea. Therefore, we had to limit the local treatment in these 2 cases to intravitreal injections. Visual recovery was significantly better in the 10 cases in which the treatment was initiated within 72 hours, as compared to the 4 cases in which it was started after more than 72 hours (*P* = 0.015).

### 4.7. Treatment

The blood-ocular barrier is similar to the blood-brain barrier and it consists of tight junctions between the endothelial cells and basement membrane of retinal capillaries and retinal pericytes. Its purpose is to protect the interior of the eye from the aggression of cells, macromolecules, and drugs, but it also prevents the entrance of antibiotic and anti-inflammatory drugs [13]. However, in posttraumatic setting, the functioning of the blood-retinal barrier is affected and therefore the penetration of systemically administered drugs is better. The direct intravitreal administration of drugs bypasses the above-mentioned barrier, but the retinal photoreceptors are sensitive to assault and these drugs may have toxic effects on the retina [13].

Clinicians often do not know the identity of the infective strain and must treat the eye empirically [1, 13]. Once culture results are available, the treatment can be modified, if necessary [1].

The most commonly used antibiotic combinations for intravitreal injections are vancomycin (1.0 mg) and amikacin (0.4 mg) or ceftazidime (2.2 mg) [13]. It has been reported that vancomycin was effective in 100% of Gram-positive endophthalmitis organisms. Regarding the Gram-negative organisms, amikacin and ceftazidime have approximately the same effectiveness, 89% [14]. Because amikacin use was associated with toxicity to retinal cells, ceftazidime is the antibiotic of choice in endophthalmitis caused by Gram-negative species. Fourth-generation fluoroquinolones, such as gatifloxacin and moxifloxacin, are effective against most ocular pathogens and have the capability to penetrate the blood-ocular barrier [13]. Therefore, we administered oral moxifloxacin orally in all our cases.

Because of the high degree of ocular inflammation associated with traumatic endophthalmitis, we used systemic steroids in 78.57% of cases. We always initiate systemic steroid therapy after 24 hours of systemic antibiotics. In the remaining 21.42% of cases, we used nonsteroidal drugs, because the inflammation was moderate.

The theoretical basis for PPV in endophthalmitis parallels a principle in general surgery: wherever pus is, it must be evacuated. The roles of vitrectomy are multiple: it eliminates a sizeable portion of germs, toxins, and inflammatory cells; it clears the media; it eliminates the vitreous scaffolding that causes traction and subsequent retinal detachment. Also, vitreous is a culture medium [1]. The intravitreal administration of antibiotics causes bacteria to lyse and release toxins. Therefore, in order to sterilize the vitreous cavity, vitrectomy is recommended to be performed 24–48 hours after the intravitreal injection of antibiotics [1]. We did not use this algorithm as the gravity of the cases compelled us to proceed to immediate PPV and injected the drugs at the end of surgery. In 5 cases, we did not perform PPV, but only intravitreal injections. The reasons were various: in 3 of the 5 cases, the opaque cornea compromised the view during surgery, making PPV impossible. All the 3 cases had a poor outcome and ended up legally blind. The other 2 cases presented within 24 hours after trauma, and we diagnosed endophthalmitis early and the visions were good: 0.4 and 0.1. Therefore, we considered that intravitreal injections alone would be enough to solve the intraocular infection. Indeed, they recovered very good visual acuities: 0.7 and 1.0, respectively (cases 11 and 14, Table 3).

**Figure 1:**
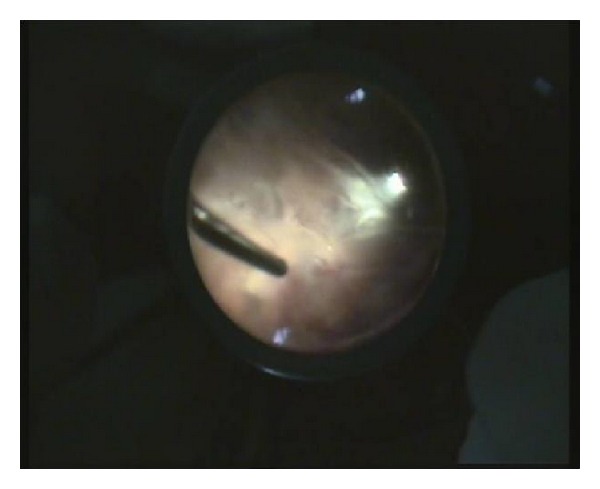
PPV in traumatic endophthalmitis.

**Figure 2:**
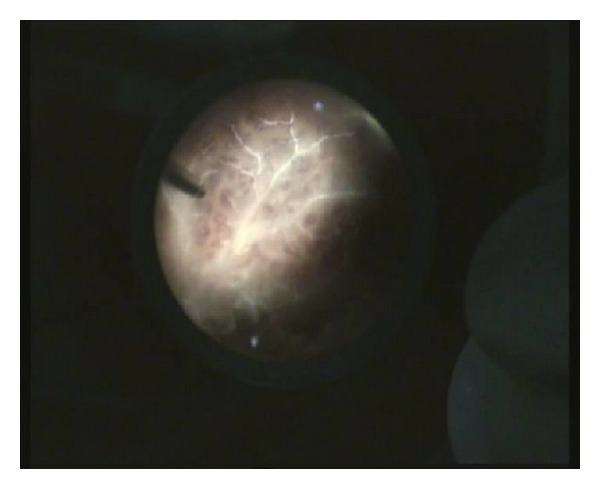
Septic retinal vasculitis and retinitis.

Traumatic endophthalmitis is a challenging indication for PPV. The difficulties come from the poor visualization generated by the hazy media and from the observation that the yellowish layers of vitreous with blood streaks can be confused with blood vessels and a detached, necrotic, nonperfused retina, as illustrated in Figure 1 (case  12, Table 3). Continuing surgery in these situations is risky, as inadvertent retinal lesions can occur, with very poor outcome. In order to prevent retinal lesions, we used a cautious, anteroposterior approach, going deep before going wide.  Figure 2 shows the same case, after having cleaned the vitreous, displaying septic retinal vasculitis and retinitis.

We did not insist on inducing the posterior vitreous detachment (PVD), as the vitreoretinal adhesion is very high in the endophthalmitis cases and the retina is necrotic and weak. Therefore, the risk of iatrogenic retinal lesions during inducing PVD is very high. In another case, visualization was disturbed and forced us to stop surgery and repeat it a few days later, once the media became clearer. The final visual outcome of this case was good, with visual acuity of 0.1 (case  2, Table 3).

### 4.8. Prophylaxis

Taking into account the severe prognosis of traumatic endophthalmitis, prophylaxis plays a major role. This comprises two paths of action in open globe injuries: intravitreal injections of antibiotics and systemic antibiotherapy. Mieler et al. administered intravitreal antibiotics in selected open globe injuries, with no case of endophthalmitis [15]. Colyer et al. [16] and Ehlers et al. [6] reported large series of open globe injuries with no intravitreal antibiotic administration and with very low rates of endophthalmitis. A multicenter clinical trial involving 346 eyes involving the intravitreal administration of gentamicin and clindamycin versus balanced salt solution control group proved lower endophthalmitis rates in the antibiotic group (0.3% versus 2.3%). However, the potential benefits must be judged in balance with the risk of retinal toxicity from aminoglycosides [8]. Regarding the prophylactic systemic antibiotics, in recent years, there was registered a shift towards the use of fluoroquinolones that have several benefits as follows: they are well tolerated and have excellent antimicrobial activity against* Bacillus* and very good penetration into the vitreous [3].

In all the open globe injuries, we administer antibiotics systemically (moxifloxacin) but not intravitreally.

### 4.9. Prognostic Factors

We identified two factors associated with a significantly worse outcome on our series: retinal detachment at presentation and delayed initiation of treatment, more than 72 hours from the debut of endophthalmitis. The outcome of traumatic endophthalmitis on our series was not influenced by age more than 50 years, initial visual acuity, the association of the IOFB, the anatomy of the wound, and the association of direct traumatic cataract and of tissue prolapse and the positive culture.

The weaknesses of this study are represented by the small number of patients and the large variability of presentations which make the cases difficult to standardize.

## 5. Conclusion

Trauma was responsible for 46.66% of all the endophthalmities that we treated for the last 5 years. Within the group of open globe injuries, the endophthalmitis rate was 8.13%. The association of an IOFB significantly increased the risk for endophthalmitis to 34.78% (*P* = 0.03). The factors associated with a worse outcome in traumatic endophthalmitis on our series were retinal detachment at presentation (*P* = 0.024) and delayed treatment with more than 72 hours from the debut of the intraocular infection (*P* = 0.015).

## Figures and Tables

**Table 1:** Traumatic endophthalmitis-ocular signs.

Wound location	
Corneal	8 (57.14%)
Scleral	6 (42.85%)
Wound size	
Below 2 mm	7 (50%)
Above/equal to 2 mm	7 (50%)
IOFB	
Yes	8 (57.14%)
No	6 (42.85%)
Direct traumatic cataract	
Yes	5 (35.71%)
No	9 (64.28%)
Ocular tissue prolapse	
Yes	4 (28.57%)
No	10 (71.42%)
Retinal detachment	
Yes	3 (21.42%)
No	11 (78.57%)

**Table 2:** Interval between endophthalmitis diagnosis and treatment.

Within 24 hrs	6 (42.85%)
>24 hrs ≤ 72 hrs	4 (28.57%)
>72 hrs ≤ 1 week	2 (14.28%)
>1 week	2 (14.28%)

**Table 3:** Synthesis of the data on our traumatic endophthalmitis series.

Case	Initial VA	Final VA (mo)∗	RD	IOFB	PPV	Delay
Case 1	hm^1^	0.08 (2)	No	No	Yes	>72 hrs ≤ 1 week
Case 2	hm	0.1 (6)	No	No	Yes	≤24 hrs
Case 3	lp^2^	hm (1)	Yes	Yes	No	>72 hrs ≤ 1 week
Case 4	lp	0.1 (15)	No	Yes	Yes	>24 hrs ≤ 72 hrs
Case 5	hm	Nlp^3^ (2)	Yes	Yes	No	>1 week
Case 6	lp	cf at 4 inch^4^ (3)	No	Yes	No	≤24 hrs
Case 7	hm	0.06 (3)	Yes	Yes	Yes	≤24 hrs
Case 8	hm	0.5 (10)	No	No	Yes	>24 hrs ≤ 72 hrs
Case 9	hm	0.1 (5)	No	No	Yes	>24 hrs ≤ 72 hrs
Case 10	0.03	0.3 (11)	No	Yes	Yes	≤24 hrs
Case 11	0.4	0.7 (5)	No	No	No	≤24 hrs
Case 12	hm	0.6 (9)	No	Yes	Yes	>24 hrs ≤ 72 hrs
Case 13	lp	0.08 (3)	No	Yes	Yes	>1 week
Case 14	0.1	1.0 (12)	No	No	No	≤24 hrs

^1^Hand motion, ^2^light perception, ^3^no light perception, and ^4^counting fingers at 4 inches.

^∗^Final visual acuity refers to the last visit, expressed in months (mo) after the acute episode.

**Table 4:** Prognostic factors on our traumatic endophthalmitis series.

Factor	VA ≥ 0.1 cases (%)	VA < 0.1 cases (%)	*P* value
Retinal detachment			
Yes	0	3 (100%)	0.024∗
No	8 (72.72%)	3 (27.27%)	
Timing			
Within 72 hours	8 (80%)	2 (20%)	0.015∗
After 72 hours	0	4 (100%)	
Age			
>50 years	3 (75%)	1 (25%)	0.640
≤50 years	5 (50%)	5 (50%)	
Initial VA			
≥0.1	2 (100%)	0	0.186
<0.1	6 (60%)	6 (50%)	
IOFB			
Yes	3 (37.50%)	5 (62.50%)	0.086
No	5 (83.33%)	1 (16.66%)	
Wound location			
Corneal	6 (75%)	2 (25%)	0.110
Scleral	2 (33.33%)	4 (66.66%)	
Wound size			
<2 mm	4 (57.14%)	3 (42.85%)	0.393
≥2 mm	4 (57.14%)	3 (42.85%)	
Cataract			
Yes	3 (60%)	2 (40%)	0.872
No	5 (55.55%)	4 (44.44%)	
Tissue prolapse			
Yes	2 (50%)	2 (50%)	0.733
No	6 (60%)	4 (40%)	
Culture			
Positive	5 (55.55%)	4 (44.44%)	0.872
Negative	3 (60%)	2 (40%)	

∗Marks the prognostic factors with statistical significance (*P* < 0.05).
